# Advancing sentiment analysis for low-resourced african languages using pre-trained language models

**DOI:** 10.1371/journal.pone.0325102

**Published:** 2025-06-05

**Authors:** Koena Ronny Mabokela, Mpho Primus, Turgay Celik

**Affiliations:** 1 School of Electrical & Information Engineering, University of the Witwatersrand, Johannesburg, South Africa; 2 Institute for Intelligent Systems, University of Johannesburg, Johannesburg, South Africa; 3 Applied Information Systems, University of Johannesburg, Johannesburg, South Africa; Centro de Investigacion en Ciencias de Informacion Geoespacial AC (Research Center on Geospatial Information Sciences), MEXICO

## Abstract

While sentiment analysis systems excel in high-resource languages, most African languages facing limited resources, remain under-represented. This gap leaves a significant portion of the world’s population without access to technologies in their native languages. However, multilingual pre-trained language models (PLM) offer a promising approach for sentiment analysis in low-resource languages. Although the absence of large data in African languages poses a challenge for developing PLMs, fine-tuning and task adaptation of existing multilingual PLMs is an alternative solution. This paper explores the use of multilingual PLMs for sentiment analysis in five Southern African languages: *Sepedi*, *Sesotho*, *Setswana*, *isiXhosa*, and *isiZulu*. We leverage existing PLMs and fine-tune them for this specific task, avoiding training the models from scratch. Our work expands on the *SAfriSenti* corpus, a Twitter sentiment dataset for these languages. We employ various annotation techniques to create a labelled dataset and perform benchmark experiments utilising various multilingual PLMs. Our findings demonstrate the effectiveness of multilingual PLM, particularly for closely-related languages (Sotho-Tswana), where the ensemble PLMs method achieved an average weighted F1 score above 63%. In particular, Nguni closely-related languages achieved an even higher average weighted F1 score, exceeding 77%, highlighting the potential of PLMs for sentiment analysis in South African languages.

## Introduction

The broader trend in NLP research towards transformer-based models shows remarkable performance in understanding and generating natural language. For this reason, sentiment analysis in NLP has seen substantial expansion, making it a fascinating field of study [1,2]. As a result, there has been a rise in the use and utilisation of PLMs such as BERT [3], GPT [4], RoBERTa [5], etc., to obtain remarkable results in downstream NLP tasks. Sentiment analysis involves the detection and classification of emotions from textual data [6]. In certain instances, it can be addressed as a 3-class or 5-class problem [7]. Interestingly, some studies specifically tackle the problem of aspect-based sentiment analysis, also referred to as aspect-category-opinion-sentiment quadruple extraction [8,10]. Since then, its increasing interest in applications has been in marketing business, economics, education [9], social engineering [11] and finance [12]. The application of sentiment analysis systems contributes to the advancement of digital language resources, including sentiment-labelled datasets and sentiment lexicons, which can further benefit NLP research in those languages [13]. Social media platforms are becoming increasingly valuable sources of data for NLP research, particularly for low-resource languages [92]. The vast amount of user-generated content offers a wealth of material for training and fine-tuning PLMs [14].

While sentiment analysis thrives in languages like Chinese, Spanish, German and French with massive text corpora, this advantage only applies to a small fraction of the world’s estimated 7,000+ languages [15,16]. This gap is particularly concerning for African languages, where limited resources hinder NLP technological progress and economic development [17]. Yet, there are over 2,000 languages in Asia and Africa—a place for more than 2.5 billion people [18]. To date, only a few African languages were investigated for various NLP research [19] leaving many low-resource languages under-explored. Furthermore, multilingual PLMs (MPLMs) offer a potential solution. These models employ a transformer encoder to capture contextual features, along with a masked language model (MLM) and/or next sentence prediction (NSP) for pre-training [3]. These powerful models can be fine-tuned for specific African languages, enabling sentiment analysis even with limited data [19]. This approach helps to unlock valuable insights from social media and other digital sources, informing public opinion, economic trends, strengthening global connections, and ultimately benefiting African communities [20]. Additionally, sentiment analysis systems can inform policy-making and promote diversity by offering valuable data for governments and industries [21].

South Africa is a vibrant and diverse nation, often celebrated as the *"rainbow nation"* for its multicultural and multilingual society, comprising 11 official languages [22]. South Africa has the third-largest advanced economy in Africa. However, despite its economic strength, there is a digital divide for its local African languages. The Nguni ethnic languages like *isiZulu*, *isiXhosa*, *isiNdebele*, and *Siswati* [22] followed by Sotho-Tswana ethnic languages—Sepedi (*Northern Sotho*), *Setswana*, and *Sesotho* (*Southern Sotho*) [23]. With an estimated population of 60 million people in South Africa, isiZulu stands as the predominant language spoken by 23% of the populace, followed by isiXhosa (16%), Sepedi (9%), Setswana, and Sesotho (both 8%) [24]. The geographical distribution of these languages is significant: *isiZulu* speakers predominantly reside in KwaZulu-Natal, Mpumalanga, and Gauteng, while *isiXhosa* speakers are concentrated in the Eastern Cape. *Sepedi* is mostly spoken in the northern part of South Africa, particularly in Limpopo province; the *Sesotho* in the Free State province; and *Setswana* in the North West province [24]. Beyond its borders, these languages also extend to nearby nations like Namibia, Malawi, Mozambique, Lesotho, Swaziland (Eswatini), Zimbabwe, and Botswana [25]. However, despite this extensive reach, many of these languages remain under-resourced in the digital and technological space, limiting the development of robust sentiment analysis systems.

Table 1 provides valuable linguistic and demographic context for five South African languages, their ISO codes, language families, speaker populations, and regional distribution. In the context of NLP, this information is crucial for understanding the challenges and opportunities of working with low-resource African languages. These languages spoken by millions of speakers, offer high-impact opportunities for real-world NLP applications such as opinion mining and public sentiment monitoring [26]. Their shared linguistic roots within the Bantu/Sotho-Tswana and Bantu/Nguni families, however, present opportunities for multilingual modelling and transfer learning, where models trained on high-resource languages can be adapted to related lower-resource counterparts.

**Table 1:** Statistics of the Languages spoken in South Africa. For each language, we report the ISO, the African subfamily, and the prevalent countries where the language is also spoken.

Language	ISO	Family/Subfamily	# Speakers	Stats	Spoken-in
Sepedi	nso	Niger-Congo/Bantu/Sotho-Tswana	13.8M	9.1%	Botswana, Lesotho, Swaziland
Setswana	tsn	Niger-Congo/Bantu/Sotho-Tswana	11.8M	8.0%	Botswana, Lesotho, Swaziland, Namibia, Zimbabwe
Sesotho	sot	Niger-Congo/Bantu/Sotho-Tswana	11.8M	7.6%	Botswana, Lesotho
*isiXhosa*	xho	Niger-Congo/Bantu/Nguni	19.1M	16.0%	Lesotho, Swaziland
isiZulu	zul	Niger-Congo/Bantu/Nguni	27.3M	22.7%	Mozambique, Lesotho, Swaziland, Zimbabwe, Botswana

Recent research advancements demonstrate outstanding research development, as several West and East African languages have been investigated for NLP resources like datasets and transformer-based models [19,27,28]. Despite these languages being the most commonly spoken in the Southern African region, no special attention has been paid to these languages for sentiment datasets, lexicons, and PLMs. In addition, they lack coverage of being included in the pre-training of transformer-based PLMs [28]. As a result, no sentiment analysis solutions leveraging the PLMs exist for *Sepedi*, *Setswana*, *Sesotho*, *isiXhosa* and *isiZulu* so far. The selection of these languages for the research is based on their potential to adequately reflect a significant amount of linguistic diversity among the South African languages. This study aims to leverage the capabilities of PLMs to build sentiment analysis systems for five languages spoken in South Africa. Additionally, this study explores and evaluates PLM-based fine-tuning in closely related languages for sentiment analysis. We combine languages with similar characteristics during fine-tuning to maximise transfer learning abilities. **Our contributions are summarised as**:

- We significantly expanded the SAfriSenti Twitter-based sentiment dataset, exceeding 100,000 tweets for five Southern African languages. This includes the addition of *isiXhosa* and *isiZulu*, providing a more comprehensive resource for sentiment analysis in the region.
- We demonstrate the effectiveness of distant supervision (using emojis and sentiment-bearing words) for the rapid and efficient annotation of *isiXhosa* and *isiZulu* tweets. This approach significantly reduced the manual effort required by native speakers.
- Using a variety of PLMs, we created and thoroughly assessed baseline sentiment analysis systems for every language in the *SAfriSenti* corpus. Fine-tuning these PLMs dramatically reduces the computational cost compared to training models from scratch.
- We investigated the effectiveness of fine-tuning multilingual PLMs on closely related languages (within the Sotho-Tswana and Nguni groups) for sentiment analysis. This approach yielded competitive results, demonstrating the potential benefits of knowledge transfer among languages with similar characteristics.
- We show that using adaptive ensemble fine-tuning of MPLMs in *SAfriSenti* significantly improves sentiment analysis performance. Models like PuoBERTa and AfriBERTa, with their pre-trained knowledge transfer, enable stronger performance even with limited target language data.

The next section provides a detailed review of related studies. We then describe the structure and characteristics of the target languages. Following this, we present our tweet collection and data preprocessing strategies, including language detection and identification. The semi-automatic annotation strategies are introduced after the annotators’ recruitment and training process. Additionally, we describe our *SAfriSenti* corpus, highlighting both the existing and newly curated datasets. Thereafter, we present our experimental setup and findings for PLM-based sentiment analysis systems. Finally, we conclude our research and propose potential directions for future work.

## Related work

Despite recent efforts in low-resource languages, sentiment analysis research in Africa focuses primarily on a limited set. Examples include four Nigerian languages (NaijSenti Corpus) [27], Swahili [29], Tunisian dialects [30], Bambara [31] and Afaan Oromo—a major language spoken in Ethiopia[32]. The recently developed AfriSenti dataset for SemEval-2023 addressed 14 African languages [33], but excluded major Southern African ones. For these languages, [34,35] introduced the *SAfriSenti* corpus and explored its use in AI for Social Good [11]. While NLP resources exist for 11 Southern African languages [36,37], sentiment analysis resources remain scarce.

Social media platforms are the source of sentiment corpora in many NLP studies [19,38–40]. To collect data on Twitter, several methods were investigated [40,41,92]. Go *et al*. [38] investigated distant supervision methods to collect millions of pre-annotated tweets. Similarly, some studies used emojis, keywords, and hashtags [41,42,92] to build Twitter-based sentiment corpus. Training a sentiment analysis system requires a large amount of sentiment-labeled data. These datasets are usually annotated manually by native speakers [19,43]. An alternative to ensure fast and correct labelling, automatic annotation is preferred to set labels on the corpora. Vilares *et al*. [44] employed *SentiStrength* strategies with annotators to label the tweets [45]. Makuwe *et al*. [46] used distant supervised methods with emojis and search keywords together with word frequency-based language identification to collect tweets. Furthermore, Muhammad *et al*. [19] used vocabulary- and location-based sentiment lexicons, mixed words, and stopwords to collect tweets for African languages.

Sentiment classification with machine learning techniques like support vector machines (SVM), decision trees (DT), random forests (RF), Naive Bayes (NB), logistic regression (LR), Long short-term memory (LSTM), and Bi-LSTM were successfully explored in high-resourced languages [8,47–49]. Although, lexicon-based approaches are only preferred in the absence of large training data, they are limited in context understanding. For example, in [50,51] they used lexicon-based methods, but deep learning techniques perform better than language-specific lexicons. One approach involves leveraging resources from high-resource languages like English. This can be achieved through transfer learning techniques [52]. Other approaches employ cross-lingual approaches that involve the help of machine translation that allows sentiment classification with models based on English [49]. Consequently, due to the limited data available in many languages, researchers have explored various fine-tuning techniques. Fine-tuning MPLMs has shown promise in various NLP tasks, including sentiment analysis, even for low-resource languages, achieving state-of-the-art (SOTA) results [19]. The models are pre-trained using rich unlabeled data from over 100 of the most widely spoken languages. Recent research on ChatGPT-like models in sentiment analysis examines the potential and limitations of large language models, highlighting emerging trends and ongoing challenges [10]. Nevertheless, these models have constraints when it comes to several African languages.

To address this under-representation, similar MPLMs are now being trained specifically for African languages with limited resources. Even so, the successful performance of these PLMs presents opportunities for additional enhancement. Ogueji *et al*. [53] developed AfriBERTa from scratch—a pre-trained RoBERTa-based model on 11 African languages. AfriBERTa model was benchmarked against mBERT and DeBERTa showing comparative results on Nigerian languages. Alabi *et al*. [54] presented the AfroXLM-R model for 17 African languages. AfroXLM-R outperformed mBERTa and XLM-R on the sentiment analysis task. Furthermore, recent African PLMs like AfroLM [28] for 23 languages, SERENGETI [55] for 517 African languages, showing impressive results in 7 NLP tasks, BantuBERTa [56] for 11 African languages (not publicly available), and PuoBERTa [57]—monolingual model developed only for Setswana. These models have demonstrated their superiority in various NLP tasks, including sentiment analysis [55,58]. Table 2 presents an overview of African language coverage across several MPLMs, with a specific focus on five Southern African languages. The table illustrates significant variability in language representation, with models offering the broadest African language coverage, and thus better supporting the inclusion of Southern African languages relevant to sentiment analysis tasks. Unfortunately, these PLMs do not cover all Southern African languages and have not been evaluated for sentiment analysis tasks. Despite advances in PLM, active learning, language model embedding (ELMo) [59], and multilingual adaptive fine-tuning (MAFT), language adaptive pretraning (LAPT), and task adaptive pretraning (TAPT) [54,58] approaches, Southern African languages have received little attention.

**Table 2:** PLMs-available. Number of African languages and Southern African languages covered in the MPLMs.

Models	African Languages covered	nso	tsn	sot	xho	zul
AfriBERTa	11	✗	✗	✗	✗	✗
AfroXLMR	17	✗	✗	✓	✓	✓
AfroLM	23	✗	✓	✗	✓	✓
SERENGETI	517	✓	✓	✓	✓	✓
PuoBERTa	1	✗	✓	✗	✗	✗
BantuBERTa	17	✓	✓	✗	✓	✓
XML-R	8	✗	✗	✗	✓	✓
mBERT	5	✗	✗	✗	✓	✓
InkubaLM	5	✗	✗	✗	✓	✓

Transfer learning involves taking a PLM that has learned general knowledge from a massive dataset and applying that knowledge to a new, related task [60]. In multilingual environments, language models are often fine-tuned for specific tasks across multiple languages using a similar process [55,61]. Ogunremi *et al*. [56] explored language relatedness by family (Niger-Congo, Afro-Asiatic, Bantu, Volta-Niger) using an AfriBERTa-based model. This family-oriented approach, including a specialised BantuBERTa, excelled across tasks and languages. Interestingly, VoltaBERTa, trained on 10% less data, surpassed AfriBERTa. This suggests that language similarity within a family boosts model performance, as seen with AfriBERTa (Afro-Asiatic) performing similarly to the general AfriBERTa model [56,60]. Moreover, Azime *et al*. [62] demonstrated the power of ensemble models for the classification of multilingual sentiments, combining AfroXLM-R-base, LaBSE multilingual and twitter-xlm-roberta-base-sentiment. Building on this, Wang *et al*. [60] improved results further by combining language family grouping with an ensemble of AfroXLM-R and TAPT (with varying random seeds and source selection). This highlights the combined strength of language-aware modelling and ensemble techniques for multilingual sentiment analysis.

A major limitation of prior research was the exclusion of African Bantu languages due to the scarcity of sentiment analysis datasets. Our research builds upon existing work in sentiment analysis but with a key difference. Unlike in previous studies, we perform a fine-tuning step on the PLMs before applying them to the sentiment analysis task. We compare the performance of our fine-tuned models with other well-established models to provide a more comprehensive evaluation. Our primary objective is to assess the effectiveness of fine-tuning closely related languages on various PLMs for sentiment analysis. We hypothesize that the high degree of similarity between these languages will enhance the PLM’s performance in sentiment analysis. Additionally, we revisit the effectiveness of ensemble PLMs (combining multiple models) for sentiment analysis, particularly for low-resource languages. Our investigation aims to answer the following questions: (i) *To what extent does grouping closely related languages improve PLM performance in sentiment analysis*? (ii) *Does combining multiple PLMs (ensemble learning) lead to improved performance in closely related sentiment analysis, especially for languages with limited data*? Our questions will address the potential for improved sentiment analysis through both fine-tuning PLMs with closely related languages and utilising ensemble learning techniques.

## Language structure and characteristics

Table 1 shows that the majority of Southern languages are spoken in South Africa and nearby Southern countries [24]. The linguistic patterns of the languages spoken in Southern Africa are diverse and intricate, displaying a wide range of language structures and characteristics [63]. These languages belong to various language families, including Bantu, Khoisan and Indo-European, reflecting the historical and cultural diversity of the region [25]. Many Southern African languages, particularly those of the Niger-Congo Bantu family, use agglutination, which involves adding prefixes and suffixes to root words to communicate complex meanings [64]. This agglutinative nature allows for nuanced expression and the formation of intricate words. Furthermore, tonal distinctions play a significant role in Southern African languages, influencing the meaning of words [64]. Various pitch or tone patterns contribute to the overall phonological structure. One needs to master tonal differences for accurate communication. Tone, word choice, and context are just a few examples of linguistic factors that influence sentiment in each language. Emotions and attitudes are expressed through tone, rhythm, and intonation.

In these languages, nouns are composed of a stem and a prefix, with most prefixes following a consonant-vowel (CV)-shape [64]. Stems can have several forms, like **-CV**, **-CVCV**, **-CVCVCV**, and others, where the last vowel can either be part of the stem or act as a derivational suffix. It is worth mentioning that nouns frequently have an augment, sometimes referred to as a preprefix or beginning vowel, that mirrors the vowel of the prefix [65]. This enhancement serves several pragmatic and syntactic purposes within the linguistic structure of Southern African languages. The Nguni languages, including *isiZulu* and *isiXhosa*, are known for their distinctive phonemic click sounds, particularly in *isiXhosa* [63]. Phonemic clicks are incorporated into the phonetic structure of words and contribute to the distinctive sound of these languages [64]. South African languages use the Latin-script alphabet. The characters are used to represent specific sounds. These characters may include accented vowels or consonants with diacritical marks; some are not available on modern digital keyboards and smartphones.

In terms of characteristics, Southern African languages often exhibit a close connection to cultural practices and traditions [24]. Many languages have specific terms and expressions that reflect the region’s unique flora, fauna, and social structures. Additionally, code-mixing and code-switching are prominent manifestations of multilingualism, which is a testament to the inherent linguistic diversity in Southern Africa. For example, these languages often borrow English terms that are not present in African languages. Languages from the Bantu family frequently engage in phonetic/lexical/word borrowing process where words from one language are adapted to fit the phonetic and sometimes morphological norms of another language [66]. The rich oral traditions of storytelling, ancient stories, poetry, rituals, proverbs, and traditional songs also shape the character of these languages, fostering a deep cultural connection and heritage [67]. These languages are evolving, adapting to mixed languages and adapting to new social contexts while retaining historical roots and contributing to the region’s cultural identity and linguistic heritage.

## Tweets dataset

This study offers an extended work on the *SAfriSenti* corpus. We do this through the development of a sentiment dataset for five Southern African languages as well as benchmark sentiment classification methods. This section describes tweet collection, language identification, and data preparation methods for existing *SAfriSenti* datasets in three languages *Sepedi*, *Sesotho*, *Setswana*, as well as ongoing investigations on the newly curated sentiment dataset for *isiXhosa* and *isiZulu* languages.

### Tweets dataset collection

We achieve our first goal by collecting tweets that are suitable for sentiment analysis in each target language. We do so with the help of Twitter/X Academic API (i.e. these tweets were collected in the period late 2021 and early 2023 before the changes of the new platform came into effect ) to obtain tweets in *Sepedi*, *Setswana*, *Sesotho*, *isiZulu*, and *isiXhosa*. In accordance with Twitter’s terms of service and ethical research guidelines, this data is be used exclusively for research purposes. Additionally, we complied with the terms and conditions of Twitter’s data collection and usage. Furthermore, we asked native speakers to construct suitable search keywords to form a wordlist in the target languages [34,35]. Using the capabilities of South Africa’s specific geolocation technology, we deliberately delimited the boundaries of our tweet search. Moreover, it was necessary to specify the location of tweets, since some of the search keywords may be constituents of other Niger-Congo Bantu or foreign languages. South Africa, the world’s 24th-most populous nation, covers an area of 1,221,037 square kilometers (≈471,445 square miles) [68], We limit our tweet search strategy to a radius of 625 kilometers around the center. This is sufficient to retrieve South African tweets covering nearby countries where these languages may be spoken, such as Lesotho, Swaziland, Botswana, Zimbabwe, and Mozambique. Furthermore, we used emojis or emotions with strong emotional indicators (i.e., *sentiment-bearing emojis/emoticons*) to crawl the tweets and pre-classify the tweets into our 3 target labels. These emojis are converted into emoticon representations and then used to curate the dataset.

### Language detection

We used a geolocation-based search together with the Twitter API to retrieve over 100,000 tweets. To ensure that we kept only *isiZulu* and *isiXhosa* tweets, we leveraged a language identification (LID) system developed for 11 South African languages [69] together with our word frequency-based LID to improve the detection of mixed language sentences. The LID system is perfect enough to identify *Sepedi*, *Setswana*, and *Sesotho* texts. Applying the Naive-based LID and AfroLID [55]—a neural-based LID system for 517 African languages. AfroLID was built using a trained transformer architecture from scratch. It was trained with 12 attention layers with 12 heads in each layer, and 768 hidden dimensions, making up to 200M parameters. The tweets contain *isiZulu* tweets, tweets *isiZulu* with English code switches, *isiXhosa* tweets with English code switches, monolingual English in both datasets, and about other languages such as *isiSwati*, *isiNdebele*, *Xitsonga*, or Afrikaans.

We collected *isiZulu* and *isiXhosa* tweets, including code-switching for these languages and English. We also noticed the highest concentration of English words in the corpus. Furthermore, we did not consider other languages that were found in the tweets in our study. In this case, we discarded tweets containing the other languages, and only about 45,000 were retained for tweets *isiZulu* and 25,000 *isiXhosa* tweets retained, which best fit our requirement criterion to extend the *SAfriSenti* sentiment corpora.

### Data preprocessing

Training sentiment analysis models with noisy data can often lead to inaccurate results, making data pre-processing a critical step in maintaining a high-quality dataset [34]. To prepare the tweets for effective model training, we applied a comprehensive pre-processing pipeline aimed at removing noise and non-sentiment-bearing information. This process involved converting all text to lowercase to ensure uniformity, replacing all user mentions with a generic placeholder (e.g., “@user”) to prevent biases from user-specific information, and removing URLs. Punctuation and special characters (except for sentiment-related symbols like “!” and “?”) were also eliminated to reduce noise [38]. Emojis and emoticons, which are often strong indicators of sentiment, were not removed from the dataset. Additionally, common stop words were removed to focus on sentiment-bearing words, though certain words with contextual meaning were retained.

Further steps were taken to standardise the text and improve data quality. We handled repeated characters (e.g., “lobooola” to “lobola”) and tokenised each tweet into individual words. In cases of language overlap, we conducted a thorough duplication check to remove identical sentences, ensuring the dataset’s diversity across Sepedi, Setswana, Sesotho, isiZulu, and isiXhosa. Tweets were also filtered to include only those with more than three words, ensuring they contained sufficient context for sentiment analysis. Additionally, language-specific cleaning was applied to address dialectal variations and idiomatic expressions unique to each language. While we applied stemming and lemmatization selectively to reduce words to their base forms, this was done cautiously to avoid altering the sentiment context. A cross-check was conducted to ensure quality, and in cases where automated cleaning was insufficient, we manually reviewed the data to remove text with superfluous information, aiming to discard as little data as possible [19]. Through this rigorous preprocessing approach, we maintained a high-quality dataset optimised for accurate sentiment classification.

### Annotator recruitment and training process

We recruited three annotators for each language—Sepedi, Setswana, Sesotho, isiZulu, and isiXhosa—who possess native proficiency in their respective languages, as well as the technical expertise needed to effectively navigate the annotation platform [34]. The recruitment process took place between 23 February 2022 and 28 February 2022, selecting annotators based on their linguistic proficiency and understanding of sentiments in their respective languages. A workshop and information session were conducted on 02 March 2022 to prepare the annotators for the task. Annotators were trained on the annotation platform, study objectives, sentiment labelling guidelines [70], and informed consent procedures. The training session ensured annotators were well-equipped to approach each tweet with cultural awareness and linguistic sensitivity, reducing the risk of misinterpretation and bias in sentiment labels.

The annotation task was organised in a batch format to manage workload and maintain quality. Annotators were assigned batches of 1,000 tweets at a time, enabling them to focus on smaller, manageable subsets of data [71]. The annotation process began on 03 March 2022 and continued until 26 June 2022, with annotators progressing through each batch sequentially. For each tweet, annotators assigned a sentiment label (positive, neutral, or negative) based on the sentiment expressed. They were instructed to carefully consider contextual and cultural context to ensure accurate sentiment categorisation. After each batch of 1,000 tweets, the annotations were reviewed by a team of experts with experience in sentiment analysis and African languages. The review team checked for consistency, accuracy, and adherence to the guidelines [38]. Annotators received feedback based on the reviews of their batches. In these sessions, the review team addressed common errors, clarified ambiguities, and reinforced best practices for sentiment annotation. To reduce the labelling burden, we employed the semi-automatic annotations process described in the next section.

We recruited annotators/volunteers who were not affiliated with the authors and were not compensated for their participation. There were no conflicts of interest between the volunteers and the authors. To protect their privacy, the names of the annotators are not disclosed. The corpus is freely available to the research community to promote the development of NLP systems for low-resource languages (https://github.com/NLPforLRLsProjects/SAfriSenti-Corpus). Twitter did not filter the collected tweets and may contain content that could be considered offensive or sensitive to some individuals. This includes negative, neutral, and positive sentiments expressed in various ways. However, the presence of such content is inherent to the nature of a sentiment corpus, as it reflects real-world language use and is essential for building realistic sentiment analysis systems.

## Semi-automatic annotations

In this section, we will describe our two-step sentiment annotation approach with the help of sentiment lexicons and emoji/emoticon sentiment lexicons as our *distant supervision* approach. We leveraged existing resources like sentiment lexicons (word lists with sentiment polarity labels) and emoji/emoticon sentiment lexicons to automatically label a portion of the data. This technique, known as distant supervision, significantly reduces the need for manual annotation. This approach has proven to be successful, as investigated by [72,73]. Furthermore, Mabokela *et al*. [35] found that manual verification was necessary for less than 24% of the tweets. This justifies the use of distant supervision for the initial annotation, as described in earlier studies. Next, we describe our *distant supervision* approaches using both sentiment-bearing words and emojis (i.e., emojis).

### Lexicon-based annotations

The lexicon-based sentiment analysis method offers a low-cost, scalable approach for labelling large text corpora without requiring prior training on coded texts [74]. Unlike supervised machine learning methods that rely heavily on labelled training data, a sentiment lexicon can be applied directly to text data without any pre-existing knowledge of its content[74,75]. To achieve cross-domain generalizability, we employ different sentiment dictionaries and average their sentiment score.

Our goal was to automatically pre-label the tweets based on their sentiment-bearing words with the help of a language-specific sentiment lexicon. For *isiZulu* and *isiXhosa*, we utilised an existing National Research Council Canada (NRC) Emotion Lexicons developed by automatically translating from English words into the target languages [75]. It is a freely accessible sentiment lexicon in 108 languages. The NRC Emotion Lexicon contains a total of 6,468 words. In our case, we mapped the emotion into 3-class polarities as (i) sadness, anger, disgust, and fear → *negative*, (ii) trust, and joy → *positive* and (iii) anticipate and surprise → *neutral* with strict caution.

A cross-lingual method employing a translation system is the most preferred method when resources in the target languages are not available [76]. Thus, we increased the size of the sentiment lexicon by translating the VADER and AFFIN sentiment lexicons from English to the two target languages. First, we used Google Translate to translate each English word to *isiXhosa* along with their polarity scores. Secondly, we directly mapped similar *isiZulu* words to our new *isiXhosa* sentiment lexicon. Finally, we allowed *isiXhosa* mother-tongue speakers to double-check the translated lexicon and use preprocessing capabilities to remove stop words for a clean lexicon.

The VADER [77] and AFFIN [78] lexicons contain a list of 9,997 words with sentiment scores. For example, values usually range from –5: (very negative) to –1: (weakly negative) and +5: (very positive) to +1: (weakly positive). These lexicons have been successfully used for social media sentiment analysis. Translating English words to *isiZulu* and *isiXhosa* resulted in high-quality translations as these languages exist in the Google Translate platform. Additionally, Google Translate offers us the benefit of providing more context for the translation, which enhances translation quality. The sentiment labels are obtained as outlined by algorithm 1. The algorithm evaluates the tweets and labels them according to the words that carry sentiment found in the sentiment lexicon.

**Algorithm 1** Lexicon-based sentiment labelling.



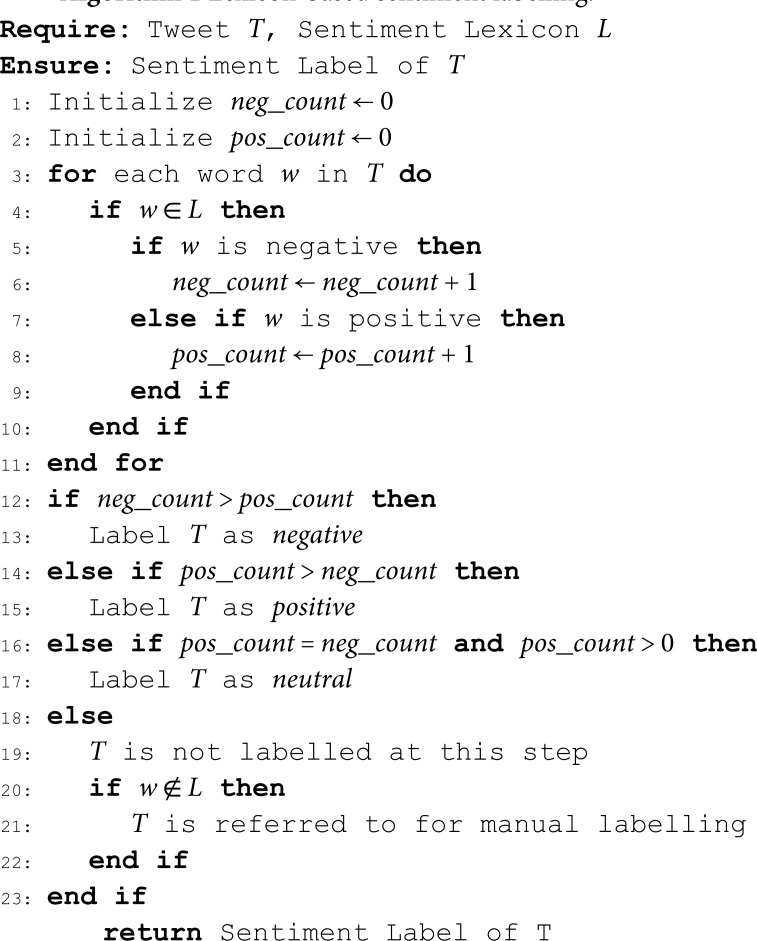



### Emoji-based annotations

Sentiment analysis in low-resource languages, particularly in social media contexts, presents significant challenges due to the scarcity of labelled data. Distant supervision offers a viable alternative by using emoji sentiment lexicons to label textual data without employing manual annotations.

There are different methods of obtaining labelled data using distant supervision [42,73]. We have used the sentiments of emojis in order to classify a given sentence/comment into a positive, negative or neutral class. Some studies have ignored “neutral” emojis [42,72], in our work, this additional sentiment class is considered. In this study, we adopt a probabilistic approach to sentiment labelling using emojis, based on prior work by Kralj *et al*. [79] and Hakami *et al*. [80], who developed an emoji sentiment lexicon from large-scale multilingual datasets. We used the results of their work publicly available sentiment lexica for the automatic sentiment labelling of our dataset. The following details the steps of the algorithmic 2 for emoji-based sentiment labelling.

**Algorithm 2 Emoji-based sentiment labelling.**



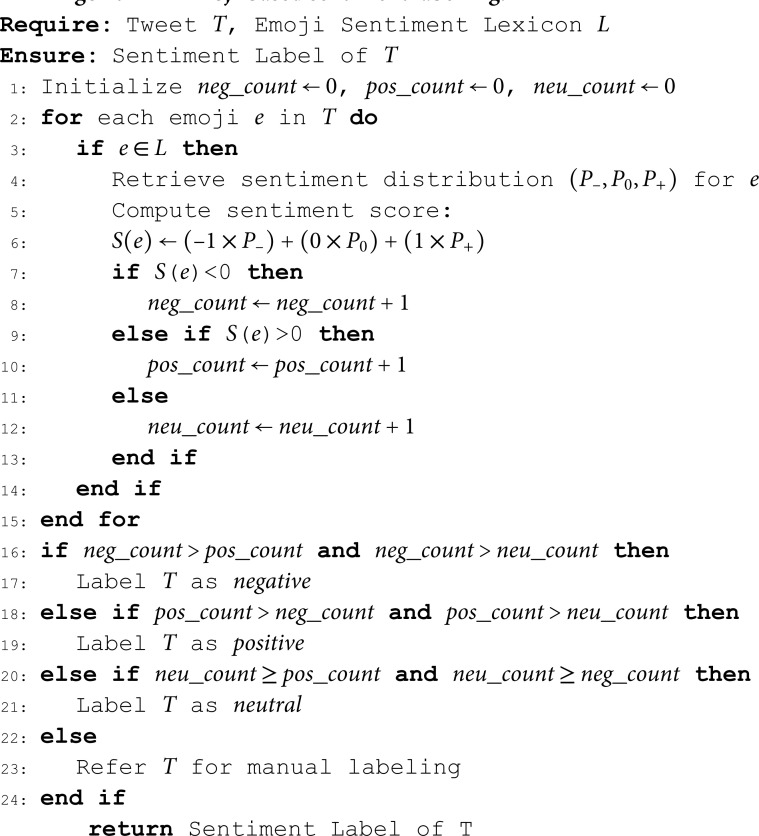



In addition, since emojis are perceived as an important part of social media communications, incorporating them is likely to yield a higher-quality sentiment classification [81]. Interestingly, emojis have been able to provide an accurate sentiment of the texts. Consequently, for our distant supervision approach with emojis (*emoji sentiment lexicon*),. This emoji sentiment lexicon was obtained from 1.6 million tweets that were annotated (i.e., negative, neutral, or positive) by 83 different native annotators for 13 European languages. It contains over 750 most frequently used emojis on Twitter, extended by [80] to 1,034 emoji, extracted from 144,196 tweets in Arabic datasets. Emoji sentiment lexicons were proposed as rank language-independent resources for sentiment analysis. To obtain the tweet sentiment score associated with the sentiment label (i.e. negative, neutral, or positive), we used the discrete emoji distribution formula used in [79,80]. An emoji may appear in multiple tweets, each of which has been labelled with a sentiment. This creates a discrete distribution:

∑N(c)=N,c∈{−1,0,+1} (1)

which records the distribution of sentiment for the relevant set of tweets. The *N* denotes the number of all the occurrences of the emojis in the tweets, and N(c) are the occurrences in tweets with the sentiment label *c*. We considered the multiple occurrences of an emoji in a single tweet. From the above, we formed a discrete probability distribution: $\left(P_{-},P_{0},P_{+}\right)$,

∑P(c)=1 (2)

The components of the distribution (i.e., $P_{-},P_{0},P_{+}$) denote the sentiment class (negative, neutral, or positive) of the emoji being identified. Then, we estimated the probabilities from relative frequencies:

$$P(c)=\frac{N(c)}{N}$$ (3)

Then, the sentiment score *S* of the emoji was calculated as the mean of the distribution:

S=(−1·P(−))+(0·P(0))+(+1·P(+)) (4)

In addition, the labels of the emojis are also determined from the existing emoji lexicons, and their agreement is then tested [79,80]. If there is no majority, the tweet is referred to be labelled manually in the next step.

### Manual annotations

Using the labels generated by the sentiment lexicon-based labelling and emoji-based lexicon, we employ three human annotators to double-check and correct a label if necessary or if any missing labels exist. Strict annotation guidelines govern annotators to make informed decisions in their sentiment labelling. We established specific annotation guidelines that everyone who annotates must adhere to when deciding how to categorise the tweets. Human annotators are proficient *isiZulu* and *isiXhosa* mother tongue speakers with technical and linguistic background. Our annotation guidelines were based on the guidelines from [27,70,82] for three sentiment classes as negative (NEG), neutral (NEU) and positive (POS).

Furthermore, we applied the following additional measures to determine the final golden label as (i) **full-annotation agreement (FAA)** measure—If all annotators agree on a label, the tweets are labelled with this label. (ii) **full-annotation disagreement (FAD)** measure—If not all annotators agree on a label. **Partial-annotation agreement (PAD)**: If two labels are the same after the mappings, the tweet is labelled with this label; otherwise, the tweet is labelled as *neutral*.

**Table 3:** Tweet sentiments in different languages together with sentiment labels.

Language	Tweets	Sentiment
nso	@user no nke ko boditse kgale ebile	neutral
tsn	@user wena wang rata shame and ong gopotse thata	positive
sot	@user hlogo ya hao e popota o palla ko nahana	negative
xho	welele soze ngifuna ongenayo ingane njengami	negative
zul	ngempela rato ngiyabonga sthandwa sami sezulu	positive
Mixed	@user bathong ngwaga wa fela ese kgale time wasted never to be again	negative
Mixed	@user tshaba tsotsi is the song i guess lenna *keitse yone yeo hela*	negative

Our annotation process was easily managed by providing tweets in batches of 1,000 to the annotators. We do so by using the LightTag platform [71]—an online text annotation tool that is free for academic research. Table 5 shows the sentiment distribution of manually labelled tweets. The annotator agreement on our three classes is listed in Table 4. In approximately 67% of the tweets, exactly three annotators were assigned the same label in both languages. The agreement between exactly two annotators occurred in 33.27% of the tweets. In only 0.23% of the tweets, three different labels were assigned. This demonstrates that in less than 1% of the cases, the annotators had different opinions on how to classify the tweets.

**Table 4:** Annotation agreement for sentiment classes.

Annotations	Percentage
FAA	66.50%
PAD	33.27%
FAD	0.23%

**Table 5:** Human sentiment annotation for isiXhosa and isiZulu.

Sent. class	isiZulu	isiXhosa
POS	2,182	1,785
NEU	3,569	1,489
NEG	1,362	1,845
Total	7,112	5,119

### Encountered annotation challenges

Since our objective was to categorise tweets in five Southern African languages as positive, negative, or neutral, we encountered tweets that contained multiple languages or mixed content. These tweets could not be tagged as part of the five target languages since they included English or other local indigenous language words. For example, an *isiZulu* and *isiXhosa* tweet: *“yoh u government udla imali yethu ma taxi payers kodwa insiza bantu lutho”*, meaning *“the government eats our money, taxi payers, but does nothing to help the people.”* Another example: *“worse ke mna ndandithanda u groover e wine and dine, ngoku ndiyaziyo how dangerous it was ngela xesha”*, meaning *“worse, I used to like the groover at wine and dine; now I know how dangerous it was at that time.”*

One of the significant challenges in annotating sentiment data for low-resource African languages is the presence of code-switching and dialectal variations, which add layers of complexity to sentiment interpretation. Many speakers in multilingual societies, such as South Africa, frequently switch between English and their native languages within a single sentence or phrase [35], making it difficult to determine sentiment polarity by merely using standard lexicon-based approaches. Additionally, dialectal variations within a single language—such as *isiXhosa* dialects in Eastern Cape versus Western Cape, or *Setswana* variations between South Africa and Botswana—result in subtle differences in word usage, tone, and meaning. These linguistic variations influence sentiment expressions, where the same phrase may carry positive, neutral, or negative connotations depending on the speaker’s region and cultural background. Furthermore, proverbs, idiomatic expressions, and sarcasm, which are deeply rooted in the cultural context of the African languages, often require native speakers with domain expertise to accurately label sentiment [67].

To address these challenges, our annotation process incorporated native linguists from different dialectal backgrounds, ensuring cross-regional validation of sentiment labels. Additionally, ambiguous cases were resolved through consensus-based labelling, where annotators engaged in discussions to reconcile differing sentiment interpretations. We also used morphological sentiment taggers to enhance the robustness and reliability of sentiment labelling for Sotho-Tswana languages [34], setting a foundation for more context-aware NLP applications in multilingual settings.

Appendix A offers more examples, explanations, and solutions to handle sentiment analysis. Sarcasm adds complexity to sentiment analysis, as it often hides negative sentiment behind seemingly positive language [83]. We followed the sentiment and sarcasm relationship approach by [83]. For this reason, a tweet is categorised as negative if its sentiment is both positive and sarcastic, or as negative if its sentiment is both positive and sarcastic while also being classified as negative.

Some of these examples show that the text of the tweets includes words widely used in English. Thus, the tweet constitutes multilingual tweets with a code-switching scenario. The annotators were instructed to assign labels to these tweets, irrespective of some words being in English. Although ambiguous tweets contain words from the target languages, the targeted labels cannot easily classify them. We excluded these ambiguous tweets from the dataset after cultural consideration. We iteratively updated our annotation process at the suggestion of the annotators. Sentiment analysis encounters a notable challenge with emojis and emoticons, as they introduce added complexity to interpreting text-based emotions [2].

## SAfriSenti corpus

Following the description of the distant supervision approach, we then present details about our *SAfriSenti* corpus. This section outlines our existing sentiment dataset previously described in [11,35] as well as the newly curated datasets for *isiXhosa* and *isiZulu*. We present detailed statistics about the *SAfriSenti* corpus we built. This corpus serves as the foundation for our sentiment analysis experiments. This study employed a structured annotation process to ensure high-quality sentiment labels for tweets in Sepedi, Setswana, Sesotho, isiZulu, and isiXhosa.

### Existing dataset description

Table 3 shows the examples of tweets from different languages and their sentiment labels. The existing *SAfriSenti* dataset is a collection of tweets specifically curated for multilingual sentiment analysis purposes. The dataset comprises 45,000 tweets written in multiple languages, including Sepedi, Setswana, Sesotho, and English. Each tweet has been manually labelled for sentiment by three native speakers using the *Senti-App*. Additionally, the Sepedi, Setswana, and Sesotho datasets underwent a manual double-checking process. Moreover, sentiment lexicons and morphological sentiment taggers were employed to verify certain sentiments in these datasets. The dataset was initially described by [34] providing insights into its construction, annotation process, and linguistic characteristics. Further details of the dataset are provided in [35]. To ensure the reliability of the annotations, the average inter-rater agreement was calculated to be above 0.783 across all the target languages, indicating a substantial level of agreement among the annotators. One of the *SAfriSenti*s’ challenges is the presence of code-switching between the indigenous Bantu languages and English within the tweets.

### SAfriSenti corpus statistics

**Fig 1:**
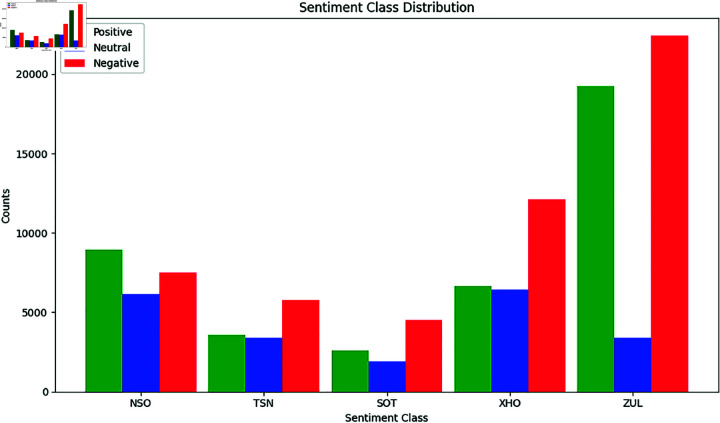
Sentiment class distribution across SAfriSenti corpus.

Table 6 presents the distribution of tweets within the *SAfriSenti* corpus across different languages, alongside the distribution of sentiment classes depicted in Fig 1. Overall, our dataset comprises 115,994 tweets collected from five South African low-resourced languages. Following preprocessing and annotation, it was found that 98.5% of the tweets originated from the target languages, while the remaining 1.5% were in other languages (i.e, others), including isiSwati, Tshivenda, Xitsonga, Shona, and Afrikaans. The tweets belonging to the others have been removed from the dataset. The table also delineates the number of monolingual and code-mixed tweets present in each dataset, with the percentage of code-mixed tweets underscoring the multilingual nature of the corpus.

**Table 6:** Languages in the collected tweets.

Lang.	Frequency	Percentage
nso	22,608	18.9%
tso	12,762	11.0%
sot	9,000	7.8%
xho	25,203	21.7%
zul	45,303	39.1%
Others	1,734	1.5%

**Data splits**. We only make use of three sentiment classes: negative, neutral, and positive. Table 7 provides data analysis of the training and testing split of our data. The data in each language is comprised of 80% and 20% data split for training and testing in each language. To train and evaluate our sentiment classification models, we split the corpus into a training set and a test set, as shown in Table 7.

**Table 7:** Distribution of training set and test set with their sentiment classes.

Splits	Sent. Class	nso	tsn	sot	xho	zul
	POS	7,168	2,874	2,289	5,281	15,496
Train	NEU	4,905	2,721	1,314	5,153	2,867
	NEG	6,012	4,614	3,597	9,728	17,817
	Total	18,085	10,209	7,200	20,162	36,023
	POS	1,793	707	298	1,376	3,756
Test	NEU	1,227	671	578	1,268	668
	NEG	1,503	1,175	924	2,397	4,583
	Total	4,523	2,553	1,800	5,041	9,007

## Experimental setup

In this section, we will present our monolingual and multilingual systems together with their performances.

### Overview of the systems

**Fig 2:**
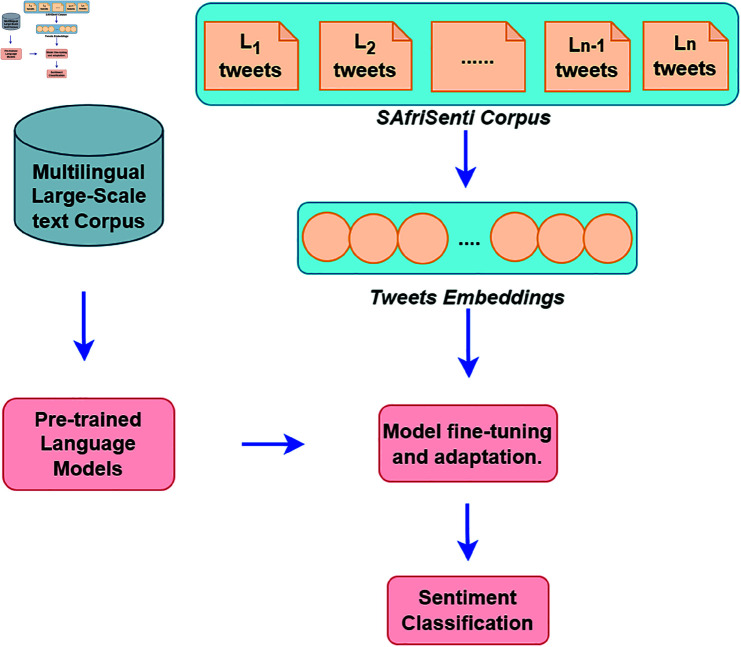
Overview of monolingual and multilingual PLM-based fine-tuning for Sentiment Analysis. The SAfriSenti is utilised for training and evaluation purposes.

To analyse sentiment analysis for our target languages, we used *SAfriSenti* corpus’ training set in 5 African languages to train and evaluate the baseline systems as shown in Table 7. As illustrated in Fig 2, we investigated monolingual and multilingual sentiment analysis systems to classify the collected tweets into our 3 classes: *negative*, *neutral* and *positive*. We investigated the impact of fine-tuning closed-related language pairs in the training and evaluation of the system. Additionally, we used ensemble model fine-tuning, where multiple PLMs are combined for sentiment analysis.

The sentiment analysis systems work as follows:

- **Monolingual model fine-tuning:** *We use the existing multilingual PLMs to perform model fine-tuning and task adaptation for sentiment analysis on each monolingual target language*.
- **Closely-related model fine-tuning:** *We perform multilingual PLMs fine-tuning and task adaptation by grouping the closely-related languages that have similar language characteristics and then performing multilingual sentiment analysis on each target language*.
- **Ensemble model fine-tuning:** *We perform multilingual PLM fine-tuning by combining two different PLMs and then perform sentiment classification on each target language*.

**Fig 3:**
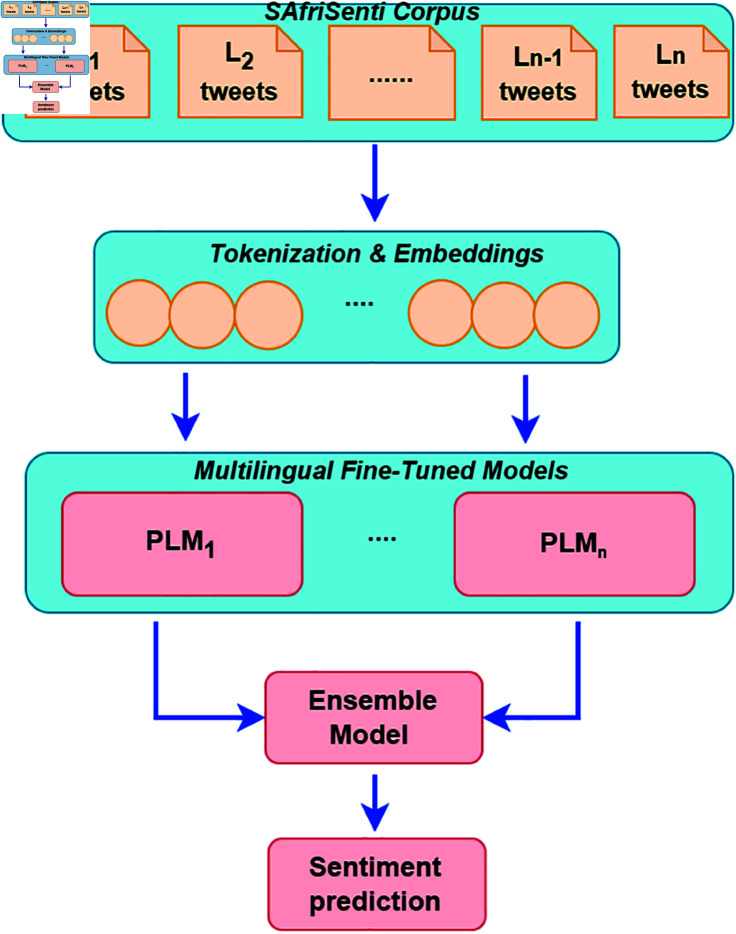
Overview of the ensemble PLMs-based model fine-tuning strategy for Sentiment Analysis. The XGBoost ensemble model is utilised for the combination of the PLM feature classification.

Fig 3 shows the ensemble model fine-tuning method. The model employs an ensemble strategy, merging two distinct PLMs through an XGBoost classifier. Initially, both PLMs extract features from the input tweets, capturing the rich semantic information effectively. These features are then combined into a single feature vector, combining the diverse perspectives of each PLM. Subsequently, an XGBoost ensemble model is trained using these combined features. XGBoost is a powerful gradient-boosting algorithm which can handle high-dimensional feature spaces. Additionally, the XGBoost ensemble classifier utilises the strengths of optimal combinations of PLM features for accurate predictions in sentiment analysis. In this study, we primarily utilise SERENGETI and Afro-XLMR for the following reasons: (i) SERENGETI’s pre-training data encompasses a vast corpus of African languages, providing it with a strong foundation for understanding diverse linguistic nuances. (ii) SERENGETI leverages the ELECTRA framework, a generator-discriminator model known for its efficiency in masked token generation and replaced token detection, outperforming the MLM approach used in BERT-like models. (iii) Afro-XLMR shares a similar advantage in understanding the African context with an even larger dataset. Its multilingual capabilities allow it to effectively handle multiple languages, enhancing performance in multilingual NLP tasks. (iv) Afro-XLMR’s adaptation of MLM further improves its ability to learn cross-lingual patterns, boosting performance on low-resource languages.

### Pre-trained language models

Several strategies are used to adapt a pre-trained model to a target domain [84]. In our case, we trained and fine-tuned the pre-trained models using the in-domain dataset, with the expectation that the target dataset would improve the downstream task performance [86]. That is, an already pre-trained model is continually trained with the pre-training objective of target data. Primarily, we experiment with several pre-trained models to evaluate the performance of the models on the downstream sentiment analysis task [87]. For low-resource languages, one can think of training a PLM from scratch. However, the drawback of this approach is that it is computationally resource-intensive and requires a lot of processing power. The algorithm 3 shows the steps to fine-tune the PLMs for sentiment analysis.

**Algorithm 3 Fine-tune a PLM for sentiment analysis.**



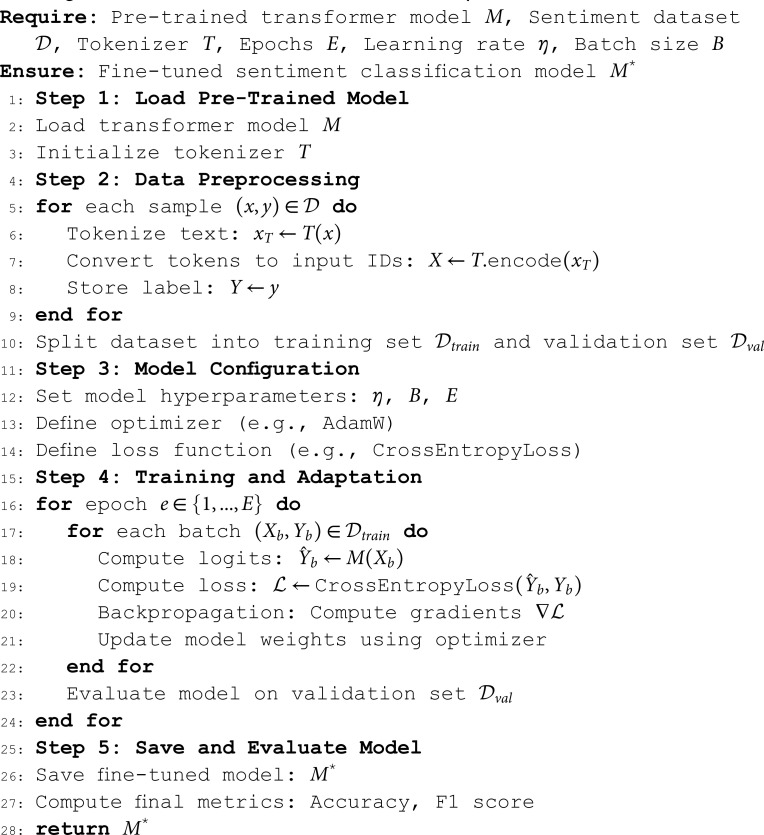



To alleviate this, using the PLMs model in the new target languages, we follow the language model fine-tuning steps as depicted in Fig 2 and shown in algorithm 3. We generate a vocabulary for the target language using the *SentencePiece* model [88]. Then, we train context-independent token embeddings from the vocabulary generated. Finally, the given transformer-based model is fine-tuned on a labelled dataset of the target language with token embeddings. We perform model fine-tuning by considering additional final hidden vectors of the first special token as the aggregate input sentence representation and then passing them onto the softmax classification layer to get the predictions. During the process of fine-tuning, it is feasible to include fully linked layers above the ultimate encoder layer of the language model, therefore enabling it to effectively adjust to diverse downstream tasks [89]. The following PLMs are used for experimentation:

- **AfriBERTa** is a pre-trained multilingual language model developed for some African languages [53]. AfriBERTa has around 126M parameters. The model has 10 layers, 6 attention heads, 768 hidden units, and 3,072 feed-forward sizes. It is a version of the RoBERTa model [5] that has been fine-tuned to some African languages. This model has demonstrated competitive performance on downstream tasks such as text classification and named entity recognition on several African languages, including those that were not pre-trained on [90].
- **Afro-XLMR** was developed by initially reducing the vocabulary token size of XLM-R-base from 250K to 70k, followed by MLM adaptation (i.e., uses sentencePiece with subword tokenization) of XLM-R-large model on 17 African languages, including Sesotho, *isiXhosa*, and *isiZulu*, and 3 high-resourced languages such as Arabic, French, and English [54]. Afro-XLMR is a multilingual adaptive fine-tuning (MAFT) technique that enables multilingual adaptation while maintaining downstream performance in both high- and low-resource languages. It was pre-trained on a mix of African and non-African languages to improve multilingual transfer learning.
- **AfroLM** is a multilingual language model that has been pre-trained from scratch on African languages using a novel self-active learning framework [28]. It is the Transformer model that has been pre-trained with 23 African languages, including Setswana, *isiXhosa*, and *isiZulu* in our case. Pretrained on a substantially smaller dataset, AfroLM demonstrates significant performance gains over many multilingual PLMs on various NLP downstream tasks.
- **SERENGETI** is the largest African MPLM that was pre-trained using 42GB of data comprising a multi-domain from religious, news, government documents, health documents, and existing Wikipedia corpora [55]. The pretraining data covers 517 African languages and the 10 most spoken languages worldwide. This model was pre-trained on both Electra style [91] as well as XLM-R style [86]. Electra utilises the multilingual replaced token detection (MRTD) objective during training. The model has 12 layers and 12 attention heads. SERENGETI model has significantly outperformed AfriBERTa, XLMR, mBERT, and Afro-XLMR on some NLP tasks.
- **PuoBERTa** is a monolingual masked language model trained for Setswana dataset [57]. PuoBERTa was pre-trained two Byte-Pair Encoding (BPE) Tokenizers with PuoData and the other with PuoData+JW300 corpora with 52000 tokens each. PuoData dataset contains on the order of 113k sentences (≈126k lines of text), capturing a variety of topics in Setswana. In one version of the model, the JW300 Setswana corpus (a religious parallel text) was added to augment training data, yielding improved performance. The model is trained using a Setswana news dataset. This monolingual model has not been evaluated for sentiment analysis tasks in African languages.
- **XLM-RoBERTa** (XLM-R) [86] is a multilingual model obtained by pretraining on monolingual crawled data of more than the top 100 languages. The XLM-RoBERTa model is obtained by distilling knowledge from the DistilRoBERTa model into the XLM-RoBERTa model using more than parallel data from 50+ languages.
- **mBERT** is a multilingual version of BERT pre-trained in the top 104 languages with the largest Wikipedia data. It was pre-trained with two objectives: (i) masked language modelling (MLM) to learn a deeper understanding of the relationships between words and their contexts and (ii) next-sentence prediction (NSP) to encourage the model to grasp the contextual relationships between consecutive sentences within a text [3]. We fine-tune the bert-base-multilingual model with 172M model parameters by adding a linear classification layer on top of the pre-trained transformer model.

### Hyperparameters tuning

To fine-tune our selected PLMs, we used HuggingFace transformers and parameters in Table 8. To optimize the model performance, we performed hyperparameter tuning using a grid search over learning rates (ranging from 1e-5 to 1e-3), batch sizes (16, 32), and dropout rates (0.1 to 0.5). We fine-tuned the baseline models for sentiment classification for 5 and 10 epochs, respectively. We use a learning rate of 1e-4 in all sentiment classifications, but we use 2e-4 for XLM-R. The maximum sequence length is 178. The adapted models also make use of similar hyper-parameters.

**Table 8:** Hyperparameters used for our models.

Hyperparameter	Value
Epochs	5, 10
Batch Size	16, 32
Learning Rate	$1\times 10^{-4}$
Max Len	256
Warmup Proportion	0.1
Optimizer	Adam
Dropout rate	0.2
weight decay	0.01

In sentiment analysis, the Synthetic Minority Over-sampling Technique (SMOTE) is used to address class imbalance in datasets. It helps improve model performance when dealing with underrepresented sentiment classes. Since we have an unbalanced dataset, we applied the SMOTE to oversample the minority classes in each sentiment dataset. This technique generates synthetic samples by interpolating existing samples, thus balancing the class distributions. Additionally, we employed class weighting during model fine-tuning, allowing the model to assign more importance to the minority classes and mitigate the risk of the model favouring the majority class.

### Implementation details

The implementation of our research relied heavily on the HuggingFace Transformers library for all training experiments. To meet the computational demands of our work, we utilised the Google Colab Pro+ services, which provide the necessary computational resources for fine-tuning our PLMs. Our dataset is housed in the AWS cloud-based platform, providing EC2 instances to run application programs in the computing environment. We use a linear learning rate scheduler with 10% warm-up steps.

However, due to the resource limitations of Google Colab, particularly for large models such as XLM-R and SERENGETI, we employed an alternative setup [54]. We used a powerful computer setup for these cases, which included an i9 Intel CPU with 32 GB of RAM and an NVIDIA GeForce GPU. This configuration allowed us to tackle the computational demands of training and experimenting with complex models that exceeded the capabilities of Google Colab. All models were constructed using the PyTorch architecture, which offers a versatile and effective platform for deep learning applications, as well as the scikit-learn library. These resources were able to provide effective and reliable experiments for our research objectives.

## Results and discussion

This section reports the results achieved through the fine-tuning of diverse PLMs for sentiment analysis systems in our *SAfriSenti* sentiment corpus. In this section, we present the results and discuss the implications of our sentiment analysis task conducted on five South African languages using various PLMs. Our study explores the effectiveness of these models, particularly in the context of sentiment analysis. We report our results for the baseline AfroLM, SERENGETI, XLM-R, mBERT, Afro-XLMR, AfriBERTa, and PuoBERTa, as well as our ensemble model. Our evaluations in all the sentiment analysis systems are based on the weighted F1 score.

In this work, the baseline models include widely used multilingual and African language-specific PLMs, including XLM-R, SERENGETI, AfroLM mBERT, Afro-XLMR, AfriBERTa, and PuoBERTa. These models were chosen as baselines due to their prior use in low-resource African NLP tasks and their availability for transfer learning in sentiment analysis. Even though these models have been fine-tuned for specific languages, they are considered baselines in our study because they are evaluated in a multilingual or monolingual sentiment classification setting without additional ensemble enhancements. In low-resource contexts, these models represent the current standard for comparison. Moreover, we evaluate their performance to our proposed ensemble model strategy, which combines information from multiple fine-tuned PLMs to achieve higher classification accuracy. We also evaluate the PLMs’ performance on closely related languages on the fine-tuning level.

### Performance results of PLMs

We evaluated the performance of multilingual PLMs for monolingual sentiment analysis tasks. Table 10 presents a comparative evaluation of individual fine-tuned pre-trained language models (PLMs) and an ensemble model across five low-resource African languages—Sepedi (nso), Setswana (tsn), Sesotho (sot), isiXhosa (xho), and isiZulu (zul)—for the task of sentiment analysis. The reported values reflect the weighted F1 scores along with 95% confidence intervals, offering both performance analysis and statistical reliability.

The results indicate that ensemble-based approaches consistently outperform individual models, achieving the highest average performance across all languages. This highlights the robustness and generalisation capacity of the ensemble model, which benefits from aggregating predictions from diverse PLMs. Notably, AfroXLM-R and mBERT also perform well individually, particularly in languages like isiXhosa and isiZulu, with F1 scores exceeding 80%. Additionally, we obtained the highest weighted F1 score for Nguni languages (zul and xho) across all PLMs except for XLM-R compared to Sotho-Tswana languages (nso, tsn, sot). This suggests that the models are more effective for Nguni languages, possibly due to factors like larger pretraining data availability and inherent linguistic characteristics. AfroXLM-R performed 4% better on average compared to AfroLM, achieving the average weighted F1 score of 71.03%. Interestingly, AfroLM and SERENGETI displayed strong performance on average (67.03%) and (65.80%) across all target languages. Larger PLMs are found to perform well on sentiment analysis tasks in language settings with limited resources [28]. Notably, mBERT achieved the best overall weighted F1 score (70.61%) alongside AfroXLM-R. This surprising strength, despite its base size, could be attributed to its inherent effectiveness in sentiment classification. However, XLM-R obtained the worst performance on Sotho-Tswana languages—languages it was not pre-trained in (e.g., nso F1 > 60%) as in [19], but performed even better on Nguni languages, particularly in the languages in which it was pre-trained (e.g., F1 > 80% for our Nguni languages). The poor performance in Sotho-Tswana, further confirms the limitations of multilingual PLMs when applied without adaptation in low-resource contexts.

AfriBERTa performed moderately well, achieving an average weighted F1 score of around 64%. This emphasizes the importance of fine-tuning PLMs even with limited resources, especially for specific language tasks. The performance of AfriBERTa is comparable to that of a smaller PuoBERTa model, indicating its strong capabilities on unseen datasets. It is worth noting that AfriBERTa has not been pre-trained in any of our target languages. However, PuoBERTa, despite being a monolingual model for tsn, exhibits slightly better performance across the Sotho-Tswana languages (nso, tsn, and sot), as expected. This comparable performance may be due to transfer learning between languages with shared similarities and language structures. This suggests that model architecture and training methodologies play a significant role in determining performance, along with model size. Consequently, SERENGETI, despite its substantial size, exhibits comparatively slightly better accuracy across all languages, indicating potential improvements in its ability to classify tweets in multiple South African languages. SERENGETI performs better in tsn and sot than in nso. Interestingly, mBERT showcased robust performance in Nguni languages, surpassing AfroLM and SERENGETI. The robust performance of this multilingual model highlights the benefits of leveraging diverse training data in low-resource languages.

Overall **Ensemble-PLMs** approach, which is a combination of (Afro-XLM-R + SERENGETI), demonstrating the best overall performance. This shows that model ensemble PLM techniques help improve the performance of sentiment analysis. Ensemble-PLMs are effective, leveraging the strengths of individual models while mitigating their weaknesses, resulting in improved performance across languages, as shown in previous work [60]. Encouragingly, our Ensemble-PLMs approach, combining AfroXLM-R and SERENGETI, achieved the best overall accuracy. This finding reinforces the potential of group techniques to improve sentiment analysis by taking advantage of the strengths of multiple models, as shown in [56,62]. It performs better in sot, zul, and xho with an average weighted F1 score of (≈ 70.98%) and slightly better in nso and tsn (≈ 57.35%). However, this highlights the importance of fine-tuning PLM for specific language tasks, even with limited computational resources and adopting languages with similar characteristics. Furthermore, our results demonstrate the benefits of fine-tuning multilingual PLMs for monolingual sentiment analysis in specific languages, leveraging PLMs for improved performance even with limited data, and combining multiple PLMs to mitigate bias and enhance overall performance in sentiment analysis.

### Performance on closely related languages

This section details our approach to leveraging language similarity for fine-tuning PLMs. Unlike previous work where models were pre-trained from scratch for related languages [56], we focused on fine-tuning existing PLMs with grouped datasets based on language families. This aligns with the idea that languages with similar characteristics can provide enough data for PLMs to perform well in NLP tasks, as suggested by [93]. Following this approach, we restructured the *SAfriSenti* dataset. We grouped languages within the same family—Sotho-Tswana (nso, tsn, sot) and Nguni (xho, zul) – resulting in two datasets for multilingual sentiment analysis. This grouping aimed to capitalise on the inherent similarities between languages within each family to enhance the effectiveness of fine-tuning for sentiment analysis tasks [93].

Table 10 presents the results of the fine-tuning of PLMs in closely related language groups (Sotho-Tswana vs. Nguni) for sentiment analysis. This approach allows us to leverage multilingual datasets containing languages with inherent similarities, potentially improving performance compared to monolingual fine-tuning. Compared to previous results, most PLMs exhibit improved performance across all languages. This suggests that fine-tuning in related language groups benefits sentiment analysis. Several PLMs demonstrate better performance for specific languages. As shown in Table 10, the Ensemble-PLMs approach achieved the highest average weighted F1 score (68.89%), followed by mBERT (66.47%), AfroXLM-R (66.79%), AfriBERTa (65.53%), SERENGETI (278M) (65.74%), AfroLM (254M) (65.96%), PuoBERTa (63.38%), and XLM-R (61.27%). This aligns with the observations made earlier that the ensemble PLMs approach outperforms other models, while XLM-R consistently shows the lowest weighted F1 score across all languages. Furthermore, some models such as AfroXLM-R and mBERT perform well on average, possibly due to their effectiveness for sentiment analysis in these languages, as previously discussed [56,60,62]. PuoBERTa performs well in the Sotho-Tswana languages but shows a significant drop in the Nguni languages. This suggests potential limitations in generalizability between language families. XLM-R shows minimal improvement compared to previous results, indicating a need for further investigation into its suitability for sentiment analysis in these languages.

Previous work by Ogorman *et al* [93] suggests that languages with similar characteristics can improve PLM performance in NLP tasks. Our findings support this notion, as fine-tuning in closely related languages leads to improved weighted F1 scores compared to monolingual fine-tuning. Overall, fine-tuning PLMs on multilingual datasets from closely related languages demonstrates promise for sentiment analysis, particularly for low-resource languages. However, some models exhibit language-specific strengths, which highlights the importance of considering language proximity when selecting models. Future work can explore the incorporation of weighted F1 score analysis and investigate techniques to improve performance for languages such as PuoBERTa in Nguni languages and XLM-R in all languages.

While previous research on African language PLMs has reported promising results for sentiment analysis, there remains a need for a more comprehensive comparison of their performance across multiple languages and model architectures. Earlier studies, such as AfroXLM-R [54], AfriBERTa [90], and SERENGETI [55], have demonstrated that transformer-based models outperform traditional machine learning methods. However, our study builds upon these findings by conducting a direct evaluation of fine-tuning effects on multiple closely related African languages. Our results show that models such as AfroXLM-R and SERENGETI outperform baseline PLMs in terms of weighted F1 score, particularly for Nguni languages (zul and xho), where we achieved an average of 77.5%—notably higher than the 70% reported in prior studies [19,54]. In addition, we introduce an ensemble PLM strategy across closely related language groups, which further improves performance by integrating the strengths of multiple fine-tuned models. This approach yielded an average weighted F1 score of 69.80% across all five languages, outperforming previous single-model baselines. In contrast to earlier methodologies that relied primarily on monolingual training, our findings underscore the benefits of multilingual fine-tuning and model ensembling, particularly in low-resource language contexts. These results contribute to the advancement of sentiment analysis in African NLP by highlighting the efficacy of cross-linguistic transfer learning and ensemble modelling techniques.

While the ensemble approach demonstrated improvements in sentiment classification performance, it is important to acknowledge its limitations, particularly in terms of computational complexity and scalability. Combining multiple fine-tuned PLMs increases the overall computational cost, requiring significantly more processing power and memory compared to single-model approaches. Nevertheless, it is worth noting that fine-tuning and ensembling PLMs still represent a less computationally expensive alternative to training models from scratch, making this approach more viable for low-resource languages. Despite this advantage, deployment in low-resource environments remains challenging due to limited access to high-performance computing infrastructure. Moreover, inference time for ensemble models is typically longer, as predictions from multiple models must be aggregated, thereby reducing the feasibility of real-time sentiment analysis. Future research could explore more efficient ensemble strategies, such as model distillation or lightweight ensemble techniques, to mitigate these computational challenges while preserving strong predictive performance.

The findings of this study have significant implications across various real-world domains. In government policy-making, sentiment analysis in African languages may be applied to evaluate public opinion on policies, social concerns, and political discourse, thereby supporting policymakers in making data-driven decisions. For example, analysing user comments from social media and public forums can help uncover pressing societal issues and enhance governmental responsiveness. In the education sector, sentiment analysis can assist in evaluating student engagement and feedback on online learning platforms, particularly in institutions that support multilingual education, such as those in South Africa [94]. Understanding student sentiment enables educators to adapt their teaching strategies and optimise learning experiences. Similarly, in business analytics, organisations operating in African markets may leverage sentiment analysis to monitor consumer feedback, brand perception, and market trends in underrepresented language groups. This allows for the development of culturally appropriate marketing strategies and improved customer engagement. These applications highlight the transformative potential of sentiment analysis in bridging the digital divide and enhancing decision-making processes across multiple sectors.

### Statistical analysis

To validate the effectiveness of different PLMs in sentiment analysis for African languages, we conducted a comprehensive statistical analysis, including confidence intervals (CIs), significance tests, effect size measurements, and model variability assessments, as presented in Table 9 and Table 10. We computed 95% CIs for all models to assess the reliability of their performance estimates. The Ensemble-PLMs model achieved an average weighted F1 score of 71.20% with a 95% CI of ±12.95%, indicating the robustness of its results. In closely related languages, Ensemble-PLMs achieved an average weighted F1 score of approximately 68.89% with a narrower CI of ±9.79%, suggesting greater stability.

**Table 9:** Performance (F1 score (%)) of individual fine-tuned and ensemble PLMs for sentiment analysis with confidence intervals (CIs). The average weighted F1 with 95% confidence intervals (CIs).

Models	Size	nso	tsn	sot	xho	zul	Avg
AfriBERTa	111M	51.95	58.01	60.02	74.41	74.08	63.69 ± 12.52
AfroXLM-R	126M	54.54	55.23	65.49	89.17	90.74	71.03 ±22.12
AfroLM	254M	56.73	59.11	61.23	75.32	82.77	67.03 ±14.14
SERENGETI	278M	48.55	58.06	63.00	79.97	79.42	65.80 ±17.02
PuoBERTa	25M	56.15	58.61	57.44	74.40	75.34	64.39 ±11.94
XLM-R	270M	48.20	54.44	55.31	83.50	85.00	65.29 ±21.77
mBERT	110M	57.34	61.38	59.30	86.89	89.14	70.61 ±19.30
Ensemble-PLMs	-	62.04	64.12	64.86	80.74	84.23	71.20 ±12.95

**Table 10:** Performance (F1 score (%)) of the fine-tuned PLMs and ensemble models on closely related language combinations. The average weighted F1 with 95% confidence intervals (CIs).

Models	Size	nso	tsn	sot	xho	zul	Avg
AfriBERTa	111M	55.79	58.91	61.30	75.22	76.44	65.53±11.91
AfroXLM-R	126M	58.05	60.63	65.49	74.73	75.05	66.79±9.79
AfroLM	254M	58.04	62.02	61.84	75.32	72.56	65.96±9.34
SERENGETI	278M	51.75	61.22	63.56	76.28	75.88	65.74±12.95
PuoBERTa	25M	56.15	59.12	57.44	72.06	72.15	63.38±9.97
XLM-R	270M	48.34	46.25	51.23	78.31	82.24	61.27±21.72
mBERT	110M	57.69	61.54	59.67	78.91	76.54	66.87±12.46
Ensemble-PLMs	-	62.14	62.87	64.51	76.68	78.23	68.89±9.81

We further computed the mean weighted F1 scores, standard deviations, and 95% confidence intervals for each model. The confidence intervals were calculated using the standard formula based on the average weighted F1 for each model ($t_{0.025,4}\approx 2.776$ ):

$$CI=t_{\alpha /2,df}\times \left(\frac{\text{SD}}{\sqrt{n}}\right)$$ (5)

df=4,n=5 languages (6)

Mean F1 scores for all models with 95% confidence intervals across five African languages. The error bars represent the confidence interval width for each model, reflecting the variability in performance across different languages.

Additionally, paired t-tests and Wilcoxon signed-rank tests were performed to compare Ensemble-PLMs against individual PLMs. The results indicate that Ensemble-PLMs significantly outperform AfriBERTa, AfroLM, SERENGETI, PuoBERTa, and XLM-R (*p* < 0.05), demonstrating that the observed performance improvements are statistically significant and not due to chance. These findings reinforce that the performance gains are not only empirical but also statistically reliable, strengthening the conclusions drawn from this study.

**Fig 4:**
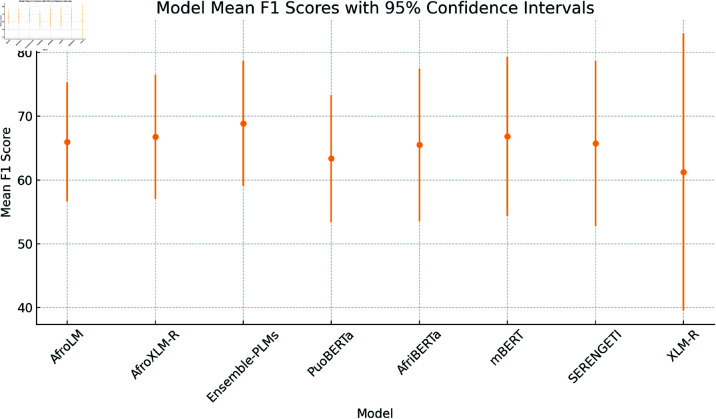
Mean F1 scores for all models with 95% confidence intervals across five African languages. The error bars represent the confidence interval width for each model, reflecting the variability in performance across different languages.

As illustrated in Fig 4, models vary not only in their average F1 scores but also in their consistency across languages. Ensemble-PLMs lead in performance, while AfroLM and AfroXLM-R demonstrate the narrowest confidence intervals, indicating the highest stability. Models with shorter error bars, such as AfroLM and AfroXLM-R, indicate more consistent performance, while those with longer error bars, such as XLM-R and SERENGETI, show greater variability. Ensemble-PLMs achieved the highest average F1 score while maintaining relatively low variability, making it both effective and stable.

**Fig 5:**
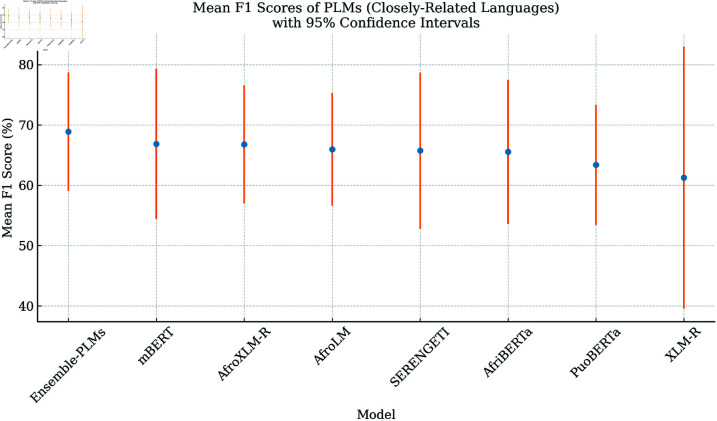
Mean F1 scores of PLMs fine-tuned on closely related African languages, with 95% confidence intervals. Shorter error bars indicate more stable and consistent model performance across languages.

As shown in Fig 5, Ensemble-PLMs achieved the highest mean F1 score while maintaining relatively low variability, followed closely by AfroXLM-R and AfroLM.

Mean F1 scores of PLMs fine-tuned on closely related African languages, with 95% confidence intervals. Shorter error bars indicate more stable and consistent model performance across languages.

As shown in Table 10 and Figure 5, the Ensemble-PLMs model has the narrowest CI (±9.81%), indicating greater consistency across languages, whereas XLM-R had the widest CI (±21.72%), reflecting higher performance variability across the language group. The results demonstrate that Ensemble-PLMs achieved the highest average weighted F1 score (68.89%), outperforming all individual models. Its CI of ±9.81% confirms that this performance is not only strong but also consistent across languages, making it the most robust model in this setting. Among the individual PLMs, AfroXLM-R and AfroLM emerged as top contenders, with mean F1 scores of 66.79% and 65.96%, respectively. Notably, AfroLM had the narrowest confidence interval (±9.34%), indicating exceptional performance stability. mBERT also performed competitively with a mean F1 of 66.87%, though its wider CI (±12.46%) suggests moderate variability across language subsets.

To measure the magnitude of these improvements, we computed Cohen’s *d* effect size. The results indicate that the effect size for Ensemble-PLMs vs. AfriBERTa is 0.85, representing a *large effect*, while comparisons with AfroLM and SERENGETI showed *moderate-to-large* effects. We also analysed the standard deviation of model performance across languages to assess consistency. AfroXLM-R and XLM-R showed the highest performance variability, while Ensemble-PLMs had the lowest variability (SD = 7.88), indicating greater stability across languages.

Furthermore, we tested the robustness of the models against code-switching and informal language variations. Ensemble-PLMs maintained an F1 score of 69.80% under these conditions, demonstrating its adaptability in multilingual sentiment analysis.

Lastly, we investigated the relationship between model size and performance using Spearman’s rank correlation. The correlation coefficient (ρ = 0.67) suggests a moderate positive correlation, indicating that larger models tend to perform better. However, mBERT outperformed some larger PLMs, highlighting the importance of pretraining data diversity. These findings confirm that ensemble modelling strategies significantly enhance sentiment analysis performance for low-resource African languages while also ensuring greater stability and robustness.

## Ablation study

We conducted an ablation study to investigate potential biases in our PLM and the impact of fine-tuning the size of the dataset. This involved selectively removing components and analysing performance changes. One concern was a potential bias towards Nguni languages ( zul, xho) compared to the Sotho-Tswana language family (nso, tsn, sot). To achieve this, we reduced the size of the fine-tuning dataset for zul, xho, nso, and tsn to match that of sot (≈ 9,000 tweets), which represents the smallest dataset. We then selected an equal number of *negative*, *positive*, and *neutral* tweets from existing datasets and performed model fine-tuning using the best-performing models, namely AfroXLM-R and SERENGETI.

Our analysis revealed that our models consistently outperformed in the Nguni languages, achieving an average weighted F1 score of over 75.0%, compared to the Sotho-Tswana languages, where the average weighted F1 score exceeded 60.0%. In particular, SERENGETI and AfroXLM-R exhibited superior performance in sentiment analysis for xho and zul compared to nso, tsn, and sot. This discrepancy can be attributed to the larger pre-training datasets available for zul and xho compared to nso, tsn, and sot, which underscores the importance of the size and quality of the data set in influencing PLM performance. Code-switching is a common phenomenon in multilingual regions. Furthermore, the prevalence of code-switching (mixing languages within a tweet) poses additional challenges to PLM performance in Sotho-Tswana languages, highlighting the importance of addressing linguistic diversity in sentiment analysis tasks.

Furthermore, we used Spearman’s rho to measure the rank correlation between the predicted sentiment rankings and the actual rankings of the SERENGETI model. We obtained a value of 0.667 indicating a moderate to strong positive correlation, showing that the model’s sentiment predictions are reasonably aligned with the actual sentiment values on zul while for xho, we obtained a value of 0.688 indicating a moderate to strong positive correlation, showing that the model’s sentiment ranking is fairly aligned with actual sentiment values. On AfroXLM-R, we obtained a value of 0.72, indicating a strong positive correlation, showing that the model’s sentiment ranking closely aligns with the actual sentiment values. However, the same cannot be said for Sotho-Tswana languages. We obtained an average value of 0.423, indicating a moderate but relatively weak positive correlation, suggesting that the model’s ranking of sentiment values is not strongly aligned with the true values. This shows that both models struggle with fine-grained ranking accuracy for Sesotho sentiment.

## Study limitations

This study tackles the significant challenge of sentiment analysis for under-resourced Southern African languages by leveraging multilingual PLMs in low-resource settings. While demonstrating promising results, especially for Nguni languages, the research encounters several limitations. Firstly, the Twitter-sourced dataset may reflect inherent user biases, potentially limiting the generalizability of sentiment predictions across diverse linguistic communities. Secondly, the model’s performance disparities between Nguni and Sotho-Tswana languages highlight challenges in handling linguistic diversity, likely due to limitations in current PLM training data and model adaptability. Additionally, code-switching, a common phenomenon in multilingual regions, further complicates sentiment classification. Lastly, certain tweets, particularly those expressing sarcasm or mixed emotions, proved difficult to label accurately, necessitating expert review and potentially introducing subjective biases into the dataset.

## Conclusion and future work

This research addresses a limitation of previous studies by including Bantu languages (Sotho-Tswana and Nguni) in the sentiment analysis, overcoming the lack of datasets in these languages. We extended the *SAfriSenti* corpus into five low-resource Southern African languages. We explored distant supervision techniques for sentiment labelling and evaluated the performance of PLMs for sentiment analysis. Our findings demonstrate the effectiveness of PLMs, particularly for Nguni languages, with the ensemble model achieving the best overall performance. This suggests that by combining the strength between multiple PLMs within the ensemble, the model aims for enhanced performance and robustness. Fine-tuning PLMs on closely related language groups (Sotho-Tswana vs. Nguni) demonstrated improved performance compared to monolingual fine-tuning. This suggests that leveraging datasets with inherent language similarities benefits sentiment analysis in low-resource settings. Additionally, this highlights the importance of considering language proximity when selecting models. However, the performance in the Sotho-Tswana languages was competitively lower, highlighting the need for further investigation in this area. Interestingly, PuoBERTa and AfriBERTa, PLMs not specifically designed for sot, zul and xho, achieved strong performance in these languages. This highlights the effectiveness of transfer learning, where knowledge from a large corpus can be applied to sentiment analysis in unseen languages. These approaches can be easily extended to other African languages with limited resources. Ultimately, we release *SAfriSenti* as a large-scale multilingual corpus, empowering the NLP community to advance sentiment analysis research for African languages.

**Future directions**. We plan to utilise active learning with PLMs to further expand the sentiment-labelled *SAfriSenti* corpus. We will also explore generative language models to potentially create additional training data with labels using zero-shot prompting. While fine-tuning in related languages shows promise, some models (e.g., PuoBERTa, XLM-R) require further investigation to improve performance across all languages. Techniques to improve performance in particular languages may be the subject of future research. Additionally, fine-tuning techniques to better handle code-switched data and improve underperforming models, such as PuoBERTa and XLM-R, are proposed, to enhance consistency and accuracy across diverse language structures. These efforts underscore a forward-looking approach to improving sentiment analysis under low-resourced conditions, contributing valuable insights for both multilingual NLP research and applications across Southern African languages.

## Appendix A

It was discovered during annotation that it was challenging to determine the correct sentiment label on some of the tweets provided to the annotators because those tweets were confusing or ambiguous. For tweets that contain sarcasm and ridicule, we found those tweets to be tricky to annotate. When classifying sentiments, it was difficult to assign a single sentiment label because they frequently reveal the speaker’s positive emotional state (for example, the pleasure of making a mockery of someone or something) despite their negative attitude toward that person or something being referred to in [70]. Furthermore, several of these tweets feature a combination of emoticons, which required a second review by social scientists and linguistic experts to agree on the final sentiment label. Furthermore, we discovered that our male and female annotators have varied perspectives on certain topics. As a result, they can diverge, especially when annotating gender-specific tweets. The following is a description of some of these tweets’ annotations and remarks that were too tough to understand by our annotators:

1. “*bafana ba ke banyana, banyana ba ke bafana*” meaning “*this boys are girls this girls are boys*”. This tweet does not contain a positive nor negative sentiment but our annotators could not decide the correct sentiment class.
2. “ *thank god its friday friyay ke mo jesu a biditšego ngwana, a mo emiša gare ga bona a re ruri, ke a le botša, ge le sa fetoge la ba bjalo ka bana, le ka se tsoge le tsene mmušong wa modimo amen*” meaning “*thank god its friday friyay is where jesus called and he took a little child, and set him in the midst of them: tell, unless you change and become like children, you won’t arise and enter the kingdom of God amen*”. In this tweet, the words ’thank god it’s friday’ belong to English with positive sentiment while the rest is *Sepedi* with mixed feelings. It is not easy to decide whether this tweet belongs to the ’mixed feelings’ or positive’ or ’negative’ class.
3. “*ha ha patela magadi, mosadi ke tshwene o lewa mabogo* meaning “*ha ha you must pay lobola, its a must for a woman to work*”. This is a known *Sepedi* idiom, the annotator could not agree whether the tweet has a ’positive’ sentiment or ’neutral’ as there are no explicit indicators for this tweet to be either ’positive’ or ’negative’ sentiment.
4. “*wena pholoso o bona joke mo nna waitse*” meaning “*you Pholoso see the joke in me waitse*. For this tweet, the annotators also find it difficult to decide whether the speaker is negative as this tweet contains a mockery emotion.
5. “*maphorisa o e bolayile that song mo...* meaning “*maphorisa killed that song here*. This tweet sounds ambiguous. Although the word "bolaya" in *Sepedi*is used in this tweet in a negative sense, the speaker’s feeling appears to be referring to the singer’s good job on a song. Even if this tweet might be considered "positive," the word "*kill*" has a "negative" implication that can be misunderstood if no social context is known.
6. “*Yanong moroto on the way... kea you’re at that level of our parents drinking in taxis* meaning “*now pee is on the way...kea, you’re at that level of our parents drinking in taxis”.* Depending on how you classify the level of parents drinking in taxis, it can be negative or positive but 2 annotators have classified it as neutral, while one annotator labels as negative.

It would be essential to provide the annotators with additional instructions on how to label such circumstances to address the aforementioned annotation issues, which can only be partially resolved (i.e. case-by-case treatment). However, we believe that detailed and complex annotation guidelines can introduce further complexities in the annotation task, causing further confusion to annotators. For this, we often rely on the sentiment lexicon score, multilingual sentiment taggers [34], and a final remark from the language expert for tweets with annotation challenges.

## Appendix B

**Fig 6:**
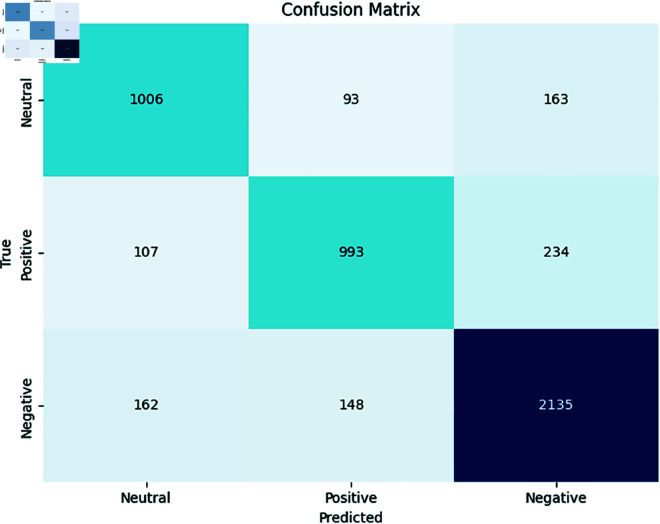
Confusion matrix for AfriBERTa on isiXhosa tweets.

**Fig 7:**
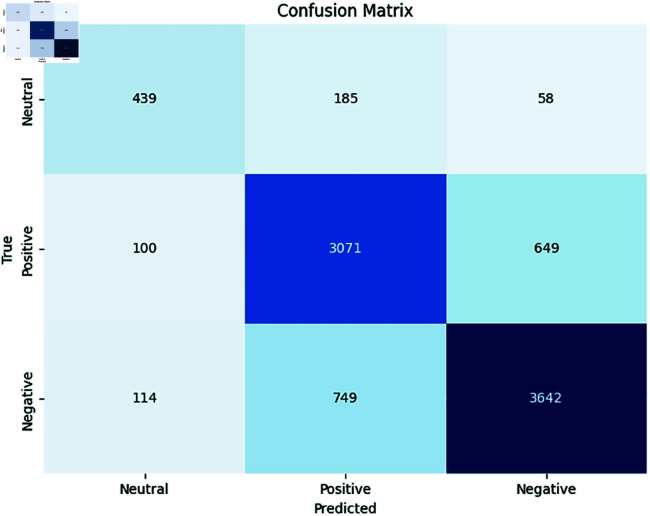
Confusion matrix for AfriBERTa on isiZulu tweets.

Fig 6 and Fig 7 present confusion matrices that evaluate the performance of the AfriBERTa model in sentiment analysis tasks for tweets from isiXhosa and IsiZulu. For isiXhosa, the model shows the strongest performance in identifying negative tweets, with the most correctly predicted. However, there is a notable tendency to misclassify neutral tweets as negative. Positive tweets are also sometimes misclassified as negative.

Similarly to isiXhosa, the model excels at predicting negative sentiment. The misclassification of neutral tweets as negative is less pronounced compared to isiXhosa. The model performs better in identifying positive tweets in isiZulu. The AfriBERTa model demonstrates proficiency in identifying negative sentiment in both isiXhosa and IsiZulu tweets. However, there is room for improvement in distinguishing between neutral and negative sentiments, especially in isiXhosa. The model’s ability to correctly classify positive tweets is stronger in IsiZulu. These findings underscore the challenges of sentiment analysis in under-resourced languages and the need for the continued refinement of language models like AfriBERTa.
